# The League of Mercy

**Published:** 1900-08-04

**Authors:** 


### The Institutional Workshop.

### THE LEAGUE OF MERCY.

### FIRST PRESENTATION OF THE ORDER.

The Presidents, Lady Presidents, Vice-presidents, and Lady Vice-presidents of the League of Mercy were entertained by their Royal Highnesses the Prince and Princess of Wales, as Grand President and Lady Grand President respectively, at Marlborough House on Friday (July 27th). The guests assembled in the gardens by noon, and shortly afterwards their Royal Highnesses (who were attended by Lady Emily Kingscote, Miss Knollys, General Sir Dighton Probyn, G.C.V.O., K.C.B., K.C.S.I., V.C., and the Hon. Sidney Greville, C.B.) arrived, and proceeded to take their places under a canopy which had been erected in the grounds. The guests were then presented in turn to their Royal Highnesses, who wore the decoration of the Order of Mercy. Those present included the following :—

Presidents.—Sir M. M. Bhownaggree, K.C.I.E., M.P., Mr. C. T. Harris, Alderman and Sheriff Sir William Treloar, Mr. Alfred H. Tarleton, Mr. J. Allen Baker, L.C.C., Colonel Arthur Allen Owen, Sir Henry Harben, Mr. A Torrance, Mr. G. J. Chatterton, the Rev. Dr. Barlow, Mr. J. B. Porter, L.C.C., Mr. W. Haydon, L.C.C., Colonel Campbell, Colonel John Davis, A.D.C., Mr. Russell Spokes, Mr. Goddard Clarke, Mr. R. C. Antrobus, Mr. T. H. W. Idris, L.C.C., Colonel R. Edis, Mr. Ambrose Pomeroy, L.C.C., the Hon. W. F. D. Smith, M.P., Mr. B. S. Straus, L.C.C., Mr. John Harris, Colonel Hughes, M.P., Sir Weetman Pearson, Bart., M.P., Mr. J. Round, M.P., Major-General Sir Henry Ewart, K C.B., Mr. Frederick Green, Mr. C. B. Hudson, M.P., Mr. Ernest Pearson, the Earl of Clarendon, the Lord Medway, the Right Hon. Sir William Hart-Dyke, Bart., M.P., the Lord Harris, G.C.S.I., G.C.I.E., Sir Fitzroy Maclean, Bart., C.B., Mr. F. S. W. Cornwallis, M.P., Mr. E. A. Hambro, Sir Joseph Sebag-Montefiore, the Most Hon. the Marquess Camden, Mr. Montague Sharpe, Mr. Thomas Lilley, Mr. H. C. Leigh Bennett, M.P., Sir J. Whittaker Ellis, Bart., Major Kingsley Foster, Mr. H. W. Keay (Mayor of Eastbourne), Mr. Edgar M. Crookshank.

Lady Presidents.—Miss Harington, the Countess Cadogan, Mrs. Dibdin, Mrs. J. Allen Baker, Mrs. Harrison, Miss Forman, Mrs. Charles Wightman, Mrs. William Cook, Mrs. Hodson, Miss Haydon, Mrs. T. Buxton Morrish, Lady Llangattock, Lady Dimsdale, Lady Burdett, Mrs. Idris, the Lady Savile, Mrs. Seymour Corkran, Mrs. Hughes, the Lady Evelyn Ewart, Mrs. McIntosh, Lady Faudel Phillips, the Lady Edith Villiers, the Lady Emily Hart-Dyke, Lady Maclean, Mrs. Davies, the Lady Edith King-Noel, the Lady Pirbright, Lady Ellis, Mrs. Tyrrell Giles.

Vice-Presidents.—Major James A. Thornhill, Dr. Henry G. Thompson and Miss Thompson, Lady Edridge, Messrs. W. R. Smith, Sidney Scholfield, Ernest Pott, G. Simmonds, E. A. Hingeston, the Rev. A. T. Wallis, Mr. J. J. Jenkins, Mrs. Leo Chapple, Miss Dando, Mr. Daniel Hines, the Rev. A. B. Boyd-Carpenter and Mrs. Boyd-Carpenter, Messrs. Maurice Cockerell, J. Oscar Drew, G. Estell, E. G. Easton, L.C.C., J. F. Flew, M. Henderson, E. H. Howe, F. Manby, Alfred Powell, W. Robertson, Arthur Williams, Mrs. Williams, Mrs. N. Cox, Miss Henniker, Mrs. Gladstone, Mrs. Stidolph, Mr. Walter R. Kersey, Mrs. Morison, Mrs. M. Sawyer, Miss Edith Giles, Miss M. A. Todd, Miss J. M. Read, Dr. Lenton Heath, Miss Nicholls, Dr. James Harper, Colonel C. A. Gorham, Messrs. R. A. Robinson, E. Spicer, D. Rennard, A. H. Poyser, J. C. Marshall, Miss G. E. Eales, Mr. J. R. Diggle, M.A., Mr. Charles Davis and Mrs. Davis, Mr. E. Chapman, Mrs. Pankhurst, Mr. Harold Hardy and Mrs. Hardy, Mr. H. J. Turner, Mr. Charles Walker, Mrs. Metcalfe, the Countess Grosvenor, Mr. and Mrs. Herbert Allingham, Mr. and Mrs. Carl Meyer, Messrs. J. Seed, H. Wright, R. Thorne Waite, Mrs. Wilkinson, Mrs. Moon, Messrs. R. T. Lidstone, E. B. Cumberland, E. De Rutzen, G. Rees, Colonel Loyd, Hon. Mrs. Halford, Mr. William Narborough, Mrs. Bishop, Mrs. Dawson, Mrs. Phillips, Colonel Richardson, R.E., Mr. W. H. Pryce, Mrs. Jackson, Miss E. T. Moore, Miss H. Smith, Mrs. Eley, Miss Randall, Miss Lowrie, Captain Oliver Young, M.P., Mrs. Young, Mrs. G. Buxton, Mrs. Wellesley Pigott, Miss Ada Palmer, Mrs. Gilbey, Mrs. Reid, Mr. J. W. Blyth, Mr. Thomas Gardner, Mr. F. W. Imbert Terry, Mrs. Reynolds, Miss E. K. Edwards, Admiral Sir Lambton Loraine, Bart., Dr. George Wilks, Mr. T. S. Johnson, Mrs. Watson, Colonel and Mrs. Ray, Mr. F. Gorell-Barnes, M.P., and Mrs. Barnes, Mr. R. G. E. Locke, Mr. Herbert Hordern, Mrs. Neame, Mr. and Mrs. Allan Tassell, Mr. Henry Payne, Mrs. Deedes, Mrs. O'Reilly, Mrs. Nettleton Balme, Mrs. Hugh Lubbock, Mr. Arthur H. Baker, Miss Laura Devas, Captain Torrens, Mr. Wheeler Bennett, Mr. J. Hamilton Fox, Mr. Samuel Page, Mr. and Mrs. A. C. Norman, Messrs. Robert A. McAndrew, J. C. Dewey, A. W. Smithers, Miss Grant, Mrs. D'Avigdor Goldsmid, the Rev. C. C. Tancock, D.D., Mr. J. Titus Barham, Mr. J. E. Lilley, Captain A. Speer, Miss Ada Long, Dr. G. C. Williamson, Mrs. Fitzroy, Mr. F. T. Aston, Mrs. Rowland Faulkner, Colonel Keays, Mr. E. T. Coppinger, Mr. and Mrs. Albert Chancellor, Mr. J. R. Hilditch, Mrs. Scott, Mr. J. D. McDougal, Lady Ommaney, Mrs. Sherrard, Miss Maclean, Mr. and Mrs. H. J. Godin, Mrs. Fleuret, Mr. C. B. Edgar, Miss Trouncer, Mr. James Wigan, Mr. J. W. Harker, Miss Trevor, Sir Charles Scotter, the Rev. W. H. Bliss, Mrs. Cave, Mr. N. Hay Forbes, Mr. M. H. Wilde, Mr. G. H. Jupp, Mr. Andrew Pears, the Rev. B. O. Sharpe, Mr. G. Gosney, Messrs. G. H. Judd, James Platford, F. S. Long, Alexander Dickson, W. Baker, S. Barrow, Mrs. Elliott, Mr. S. H. Valentine, Mr. W. Harris, Mrs. Kimber, Colonel Grubb, Mrs. Horan, Mr. Stuart Gordon, Captain Gunton, Miss G. L. M. Elliott, Mr. W. A. Browne, Mr. A. B. Fisher, Mrs. Collins, Mrs. John Harris, Sir Henry Burdett, K.C.B. (treasurer), the hon. secretaries, Lord Wolverton, Dr. W. J. Collins, and Mr. J. Harrison, and Colonel Knollys (registrar) and Mrs. Knollys.

After the reception of the guests the Prince and Princess of Wales, in accordance with the statutes of the League, presented the Order of Mercy for services to the League, and also for services in connection with the London hospitals, to the following: Sir Whittaker and Lady Ellis, Mr. E. A. Hambro, and Mrs. Herbert Allingham, the ladies receiving their decorations from the hands of the Princess and the gentlemen theirs from the Prince.

Two more orders have been awarded, but the presentation of them was deferred owing to the unavoidable absence of the lady presidents (the Duchess of Westminster and Isabella Lady Lennard), to whom they were to have been given.

